# DAPK3 is Essential for DBP‐Induced Autophagy of Mouse Leydig Cells

**DOI:** 10.1002/advs.202413936

**Published:** 2025-03-06

**Authors:** Si Yang, Ying Yang, Linlin Xu, Chaoju Hao, Jiaxiang Chen

**Affiliations:** ^1^ Department of Physiology School of Basic Medical Sciences Jiangxi Medical College Nanchang University Nanchang 330006 P. R. China; ^2^ Huankui Academy Jiangxi Medical College Nanchang University Nanchang 330006 P. R. China; ^3^ Department of Pathology The First Affiliated Hospital Jiangxi Medical College Nanchang University Nanchang 330006 P. R. China; ^4^ Library Jiangxi Medical College Nanchang University Nanchang 330006 P. R. China

**Keywords:** autophagy, DAPK3, dibutyl phthalate, leydig cells

## Abstract

Dibutyl phthalate (DBP) has been widely used in the manufacture of various daily and industrial products. As one of the most important endocrine disruptors, DBP has male reproductive toxicity and can lead to testicular dysfunction. In view of the fact that Leydig cells are important functional and structural units in the testis, their damage will affect testicular function. However, the underlying mechanism of DBP‐caused damage to mouse Leydig cells remains elusive. In the study, it is confirmed that DBP can promote the expression of death‐associated protein kinase 3 (DAPK3), thereby inducing autophagy of mouse Leydig cells by using in vivo and in vitro experiments. Also, bioinformatics analysis and molecular biology experimental techniques are utilized to further demonstrate that DBP‐induced upregulation of DAPK3 results from both the activated transcription by specific protein 2 (Sp2) and the decreased ubiquitination and degradation by parkin RBR E3 ubiquitin‐protein ligase (PRKN). Interestingly, melatonin can inhibit both Sp2/DAPK3 and PRKN/DAPK3 signaling pathways by inhibiting oxidative stress, thereby alleviating DBP‐induced autophagy of mouse Leydig cells. Overall, the study unravels a novel regulatory mechanism of DBP‐induced autophagy of mouse Leydig cells and identifies DAPK3 as a potential therapeutic target for DBP‐caused damage to the male reproductive system.

## Graphical abstract

This study has confirmed that death‐associated protein kinase 3 (DAPK3) is essential for dibutyl phthalate (DBP)‐induced autophagy of mouse Leydig cells, and is regulated by both transcription factor specific protein 2 (Sp2) and parkin RBR E3 ubiquitin‐protein ligase (PRKN), and melatonin can alleviate DBP‐induced autophagy of the cells.

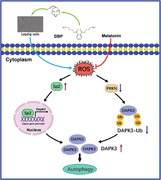

## 1 Introduction

Dibutyl phthalate (DBP) is one of the most widely used plasticizers in the manufacturing of medical devices, food containers, cosmetics, and children's toys.^[^ ^1^ ^]^ Due to its non‐covalent bonding with plastic substrates, DBP is easily released from plastics, resulting in its widespread presence in the environment, which can enter the body through skin contact, and respiratory and dietary pathways.^[^ ^2^ ^]^ DBP has been detected in water,^[^ ^3^ ^]^ soils^[^ ^4^ ^]^ and air^[^ ^5^ ^]^ with concentration ranges of 1.0–13.5 µg L^−1^, 2.75–29.37 mg kg^−1^ and 12.9–89 ng m^−3^, respectively. The accumulation of DBP and its metabolite monobutyl phthalate (MBP) can also be detected in human hair, urine, serum, semen, breast milk, saliva, and nails.^[^ ^6^, ^7^, ^8^ ^]^

As one of the endocrine disrupting chemicals (EDCs), it has been reported that DBP can interfere with the development and maturation of male reproductive organs, and results in a series of irreversible damage to testicular cells.^[^ ^9^ ^]^ Epidemiological research has indicated a close connection between DBP exposure and poor sperm quality.^[^ ^10^ ^]^ Prenatal DBP exposure can cause the dysfunction of Leydig cells in the male offspring, followed by testosterone synthesis disorders and reproductive function impairment.^[^ ^11^, ^12^, ^13^ ^]^ Leydig cells are located in the interstitial compartment and play an essential role in regulating spermatogenesis due to the function of synthesizing and secreting testosterone.^[^ ^14^, ^15^ ^]^

Many kinds of chemicals such as di‐2‐ethylhexyl phthalate (DEHP), bisphenol A (BPA), and tri‐ortho‐cresyl phosphate (TOCP) can induce autophagy of mouse Leydig cells.^[^ ^16^, ^17^, ^18^ ^]^ Autophagy is crucial in the process of spermatogenesis and can lead to spermatogenesis arrest by affecting testosterone production and the metabolism of androgen‐binding proteins.^[^ ^19^ ^]^ DBP‐induced endoplasmic reticulum (ER) stress triggers autophagy of mouse spermatocyte‐derived germ cells and GC‐2 cells.^[^ ^20^, ^21^ ^]^ We have previously found that DBP can cause testis tissue damage and spermatogenesis arrest in mice by causing Leydig cell damage.^[^ ^22^ ^]^ However, it remains poorly understood whether DBP can induce autophagy of Leydig cells and its potential mechanism.

Death‐associated protein kinase 3 (DAPK3) is a member of the DAPK family consisting of DAPK1, DAPK2, DAPK3, DRAK1, and DRAK2.^[^ ^23^ ^]^ Knockdown of DAPK3 in skeletal muscle tissue of starved mice blocks starvation‐induced phosphorylation of Beclin 1 at serine 90, suggesting a close relationship between DAPK3 and autophagy.^[^ ^24^ ^]^ The expression of transcription inhibitory factor death domain‐associated protein 6 (Daxx) is significantly upregulated in prostate tumor cells, and inhibits autophagy by reducing the expression of DAPK3 and Unc‐51‐like kinase 1 (ULK1), and ultimately promoting the tumorigenicity of prostate cancer cells.^[^ ^25^ ^]^ Nevertheless, it needs to be further investigated whether DAPK3 is involved in DBP‐induced autophagy of mouse Leydig cells.

This study aims to investigate the role and mechanism of DAPK3 in DBP‐induced autophagy of mouse Leydig cells. Herein, we have identified for the first time that DAPK3 is a key driver of DBP‐induced autophagy of mouse Leydig cells and the transcription of the *Dapk3* gene can be promoted by transcription factor‐specific protein 2 (Sp2). Interestingly, DBP can also inhibit the binding of DAPK3 with parkin RBR E3 ubiquitin‐protein ligase (PRKN), an E3 ubiquitin ligase, and subsequently inhibit its ubiquitination and degradation to evoke autophagy. Meanwhile, melatonin has been confirmed to inhibit oxidative stress and Sp2/DAPK3 and PRKN/DAPK3 signaling pathways and alleviate DBP‐induced autophagy of mouse Leydig cells. Our findings not only reveal a novel mechanism of DBP‐induced autophagy of mouse Leydig cells but also provide a theoretical basis for identifying DAPK3 as a potential therapeutic target for preventing Leydig cell injury.

## 2 Results

### 2.1 DBP Induces Autophagy of Mouse Leydig Cells

**Figure 1:**
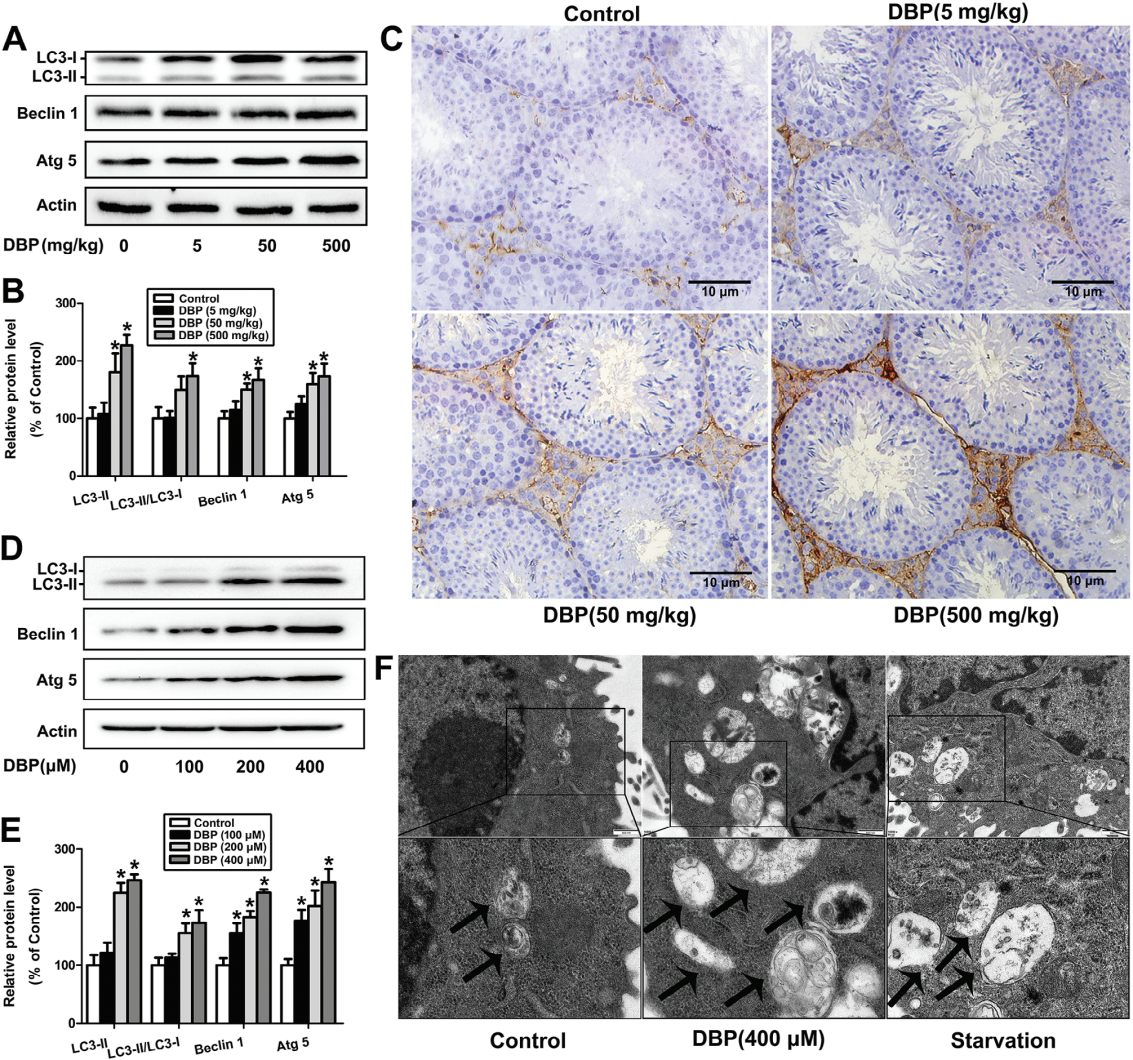
DBP induces autophagy of mouse Leydig cells. A,B) The protein levels of LC3, Beclin 1, and Atg 5 in the testis tissue were determined by Western blot after male mice were treated with 0–500 mg kg^−1^ DBP for 28 d. n = 6. C) The expression of LC3B in the testis tissue was determined by immunohistochemistry. n = 6. Scale bar = 10 µm. D,E) The protein contents of LC3, Beclin 1, and Atg 5 were determined after TM3 cells were exposed to 0–400 µm DBP for 24 h. n = 3. F) TEM showed an increased number of autophagic vacuoles in the 400 µm DBP‐treated cells. n = 3. The autophagic vacuoles were marked with black arrows. Scale bar = 500 nm. *P < 0.05. Data are represented as means ± SEM. ^*^ *p* < 0.05, by one‐way ANOVA with LSD method (B, E).

In our previous study, we have proved that the autophagy pathway is significantly affected in the DBP‐treated mouse Leydig cells according to RNA‐sequencing analysis.^[^ ^22^ ^]^ In order to further elucidate whether DBP induces autophagy and its potential mechanism, the autophagy level in the testis tissue was first detected after male mice were exposed to 0, 5, 50, and 500 mg kg^−1^ DBP for 28 d. As can be seen from **Figure** **1**A,B, DBP increased the LC3‐II/LC3‐I ratio and the protein levels of autophagy‐related proteins LC3‐II, Beclin 1, and Atg 5 in the testis tissue (*P* < 0.05). Interestingly, DBP especially increased the expression of LC3B in the Leydig cells of the testis tissue (Figure 1C). Subsequently, TM3 cells were set as an in vitro cell model and treated with 0, 100, 200, and 400 µm DBP for 24 h.^[^ ^22^ ^]^ DBP was shown to significantly increase the LC3‐II/LC3‐I ratio, the contents of LC3‐II, Beclin 1, and Atg 5, as well as the number of autophagic vesicles in TM3 cells (*P* < 0.05, Figure 1D–F). Furthermore, the inhibition of cell viability caused by DBP could be alleviated by 3‐methyladenine (3‐MA) (*P* < 0.05, Figure S1A, Supporting Information). Compared with the DBP‐treated group, the expression level of autophagy‐related proteins and the number of autophagic vesicles were significantly reduced in the 3‐MA plus DBP‐treated group (*P* < 0.05, Figure S1B–D, Supporting Information). These results confirm that DBP can induce autophagy of mouse Leydig cells.

### 2.2 DBP Promotes the Expression of DAPK3 in Mouse Leydig Cells

**Figure 2:**
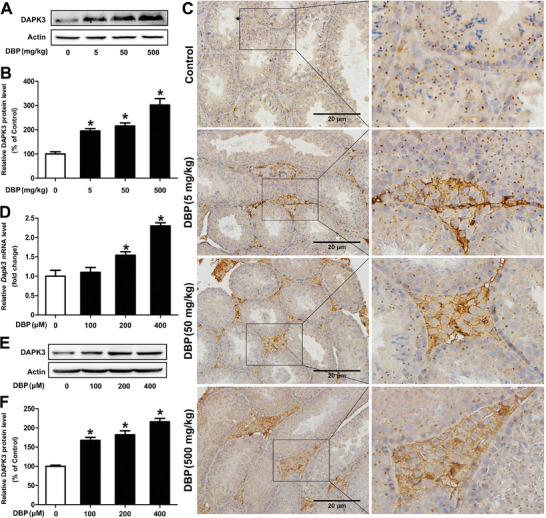
DBP promotes the expression of DAPK3 in mouse Leydig cells. A–C) After adult male mice were administrated with 0–500 mg kg^−1^ DBP for 28 d, the expression of DAPK3 in the testis tissue was detected by Western blot and immunohistochemistry, respectively. n = 6. D–F) The mRNA and protein levels of DAPK3 were determined after TM3 cells were exposed to 0–400 µm DBP for 24 h. n = 3. Data are represented as means ± SEM. *P < 0.05, by one‐way ANOVA with LSD method (B, D, and F).

To discover the potential mechanism of DBP‐induced autophagy of mouse Leydig cells, we further analyzed the differentially expressed genes (DEGs) related to autophagy through qPCR assay and found that the mRNA level of *Dapk3* was significantly upregulated in the DBP‐treated cells (*P* < 0.05, Figure S2, Supporting Information). Subsequently, DBP was identified to promote the expression of DAPK3 in both testis tissue and TM3 cells, and the increased expression resulted mainly from Leydig cells rather than germ cells (*P* < 0.05, **Figure** **2**). These results indicate that DBP can promote the expression of DAPK3 in mouse Leydig cells.

### 2.3 DAPK3 is Essential for DBP‐Induced Autophagy of Mouse Leydig Cells

**Figure 3:**
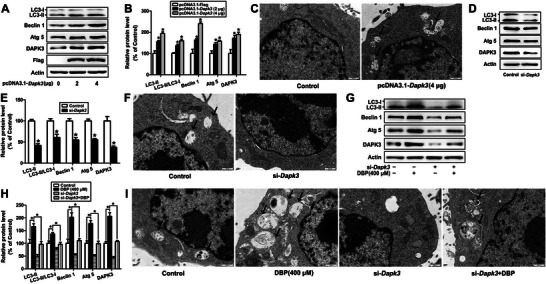
DAPK3 is essential for DBP‐induced autophagy of mouse Leydig cells. A–F) TM3 cells were transfected with 0, 2, and 4 µg pcDNA3.1‐*Dapk3* for 48 h, or 250 pmol µL^−1^ si‐*Dapk3* for 24 h, and the protein levels of DAPK3, LC3, Beclin 1 and Atg 5, as well as the numbers of autophagic vacuoles were detected. G–I) TM3 cells were treated with 0 or 400 µm DBP for 24 h in the presence or absence of 250 pmol µL^−1^ si‐*Dapk3*, and the protein levels of LC3, Beclin 1, and Atg 5, as well as the numbers of autophagic vacuoles, were determined. Data are represented as means ± SEM, n = 3. *P < 0.05, by one‐way ANOVA with LSD method (B, H) or independent sample *t*‐test (E).

To investigate whether DAPK3 is essential for DBP‐induced autophagy of mouse Leydig cells, the autophagy level in response to the overexpression or knockdown of DAPK3 in TM3 cells was first determined. As shown in **Figure** **3**, DAPK3 overexpression increased the protein levels of LC3‐II, Beclin 1, and Atg 5, and the ratio of LC3‐II/LC3‐I, as well as the numbers of autophagic vacuoles (*P* < 0.05), while knockdown of DAPK3 significantly inhibited autophagy of the cells (*P* < 0.05). Furthermore, the induction of autophagy by DBP could be reduced by DAPK3 depletion (*P* < 0.05). These results demonstrate that DAPK3 is essential for DBP‐induced autophagy of mouse Leydig cells.

### 2.4 Transcription of Dapk3 Gene is Promoted by Sp2

**Figure 4:**
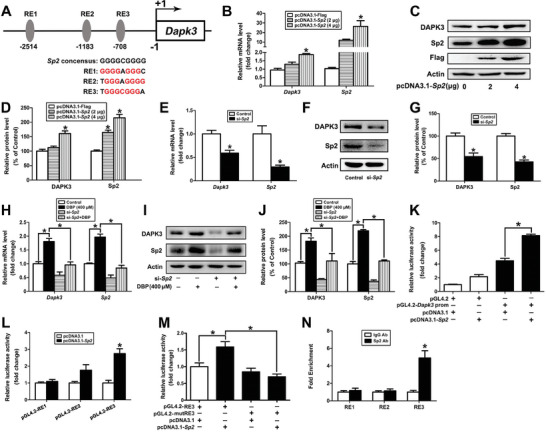
Transcription of the *Dapk3* gene is promoted by Sp2. A) Three potential REs of the *Dapk3* gene were predicted by the JASPAR database. B–G) TM3 cells were transfected with 0, 2, and 4 µg pcDNA3.1‐*Sp2* for 48 h or 250 pmol µL^−1^ si‐*Sp2* for 24 h, and the mRNA and protein levels of DAPK3 were determined. H–J) After TM3 cells were exposed to 0 or 400 µm DBP for 24 h in the presence or absence of 250 pmol µL^−1^ si‐*Sp2*, the mRNA and protein contents of DAPK3 were detected. K) After TM3 cells were transfected with pcDNA3.1 or pcDNA3.1‐*Sp2* in the presence or absence of pGL4.2‐*Dapk3* prom, the luciferase activity was detected. L) TM3 cells were transfected with pGL4.2‐RE1, pGL4.2‐RE2, or pGL4.2‐RE3 together with or without pcDNA3.1‐*Sp2*, the luciferase activity was then determined. M) After TM3 cells were transfected with pcDNA3.1 or pcDNA3.1‐*Sp2* in the presence of pGL4.2‐RE3 or pGL4.2‐mutRE3, the luciferase activity was determined. N) The binding of Sp2 to the RE3 site of the *Dapk3* gene was verified by ChIP‐qPCR. Data are represented as means ± SEM, n = 3. *P < 0.05, by one‐way ANOVA with LSD method (B, D, H, J, K, and M) or independent sample *t*‐test (E, G, L, and N).

To investigate the potential mechanism of DAPK3 upregulation, we analyzed the promoter region of the *Dapk3* gene by using the JASPAR website. Interestingly, there were three putative Sp2‐responsive elements (REs) in the promoter of *Dapk3* gene located at −2514–−2506 (RE1), −1183–−1175 (RE2), and −708–−700 (RE3), respectively, indicating that *Dapk3* gene might be transcriptionally regulated by Sp2 (**Figure** **4**A). Subsequently, overexpression of Sp2 was found to upregulate the expression of DAPK3 at both the mRNA and protein levels; while Sp2 depletion suppressed DAPK3 expression (*P* < 0.05, Figure 4B–G). Additionally, DBP‐induced upregulation of DAPK3 could be rescued by Sp2 knockdown (*P* < 0.05, Figure 4H–J). Finally, Sp2 was identified to bind to the RE3 site of the *Dapk3* gene and promoted its gene transcription according to the results of ChIP‐qPCR and dual‐luciferase reporter assays (Figure 4K–N). Taken together, these results suggest that Sp2 can promote the transcription of the *Dapk3* gene in mouse Leydig cells.

### 2.5 Sp2 is Involved in DBP‐Induced Autophagy of Mouse Leydig Cells

**Figure 5:**
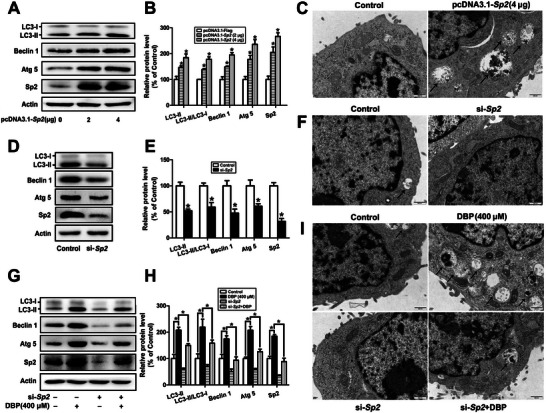
Sp2 is involved in DBP‐induced autophagy of mouse Leydig cells. A–F) The protein levels of LC3, Beclin 1, and Atg 5 and the amount of autophagic vesicles were determined after TM3 cells were transfected with 0, 2, and 4 µg pcDNA3.1‐*Sp2* for 48 h or 250 pmol µL^−1^ si‐*Sp2* for 24 h, respectively. G–I) The protein levels of LC3, Beclin 1, and Atg 5 and the amount of autophagic vesicles were detected after TM3 cells were exposed to 0 or 400 µm DBP with or without Sp2 knockdown. Data are represented as means ± SEM, n = 3. *P < 0.05, by one‐way ANOVA with LSD method (B, H) or independent sample *t*‐test (E).

To confirm the role of Sp2 in DBP‐induced autophagy of mouse Leydig cells, a series of experiments were performed. First, Sp2 overexpression was shown to enhance the protein levels of LC3‐II, Beclin 1, and Atg 5, and the ratio of LC3‐II/LC3‐I, as well as the amount of autophagic vesicles (*P* < 0.05, **Figure** **5**A–C). Afterward, Sp2 depletion was found to significantly decrease the levels of autophagy‐related proteins and the number of autophagic vesicles (*P* < 0.05, Figure 5D–F), suggesting that Sp2 can induce autophagy of TM3 cells. Finally, the knockdown of Sp2 could reverse DBP‐induced autophagy of TM3 cells (*P* < 0.05, Figure 5G–I). Taken together, these results demonstrate that the Sp2/DAPK3 signaling pathway plays an essential role in DBP‐induced autophagy of mouse Leydig cells.

### 2.6 DBP Inhibits Ubiquitin–Proteasomal Degradation of DAPK3

**Figure 6:**
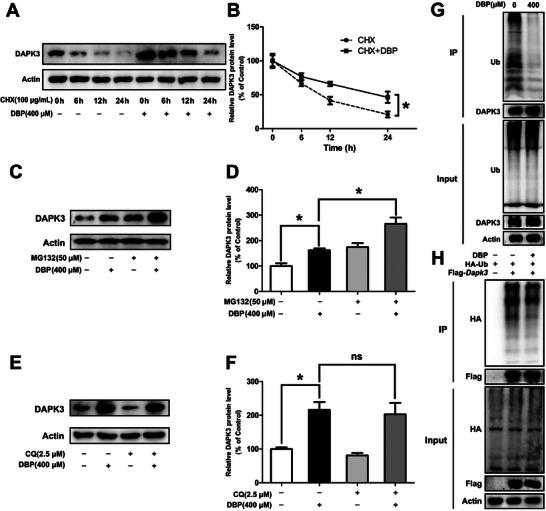
DBP treatment inhibits ubiquitin–proteasomal degradation of DAPK3 in mouse Leydig cells. A,B) The protein level of DAPK3 was analyzed after TM3 cells were exposed to 100 µg mL^−1^ CHX for 0, 6, 12, and 24 h with or without 400 µm DBP. C–F) The protein level of DAPK3 was detected after TM3 cells were treated with 0 or 400 µm DBP for 24 h with or without 50 µm MG132 for an additional 8 h, or 2.5 µM CQ for an additional 6 h. G) TM3 cells were treated with 0 or 400 µm DBP for 24 h and then exposed to 50 µM MG132 for 8 h, the ubiquitylation of DAPK3 was detected by Co‐IP assay using anti‐Ub antibody. H) TM3 cells were transfected with indicated plasmids for 48 h with or without 400 µm DBP for 24 h, and then 50 µm MG132 was added for 8 h, the ubiquitylation of DAPK3 was detected by Co‐IP assay using anti‐HA antibody. Data are represented as means ± SEM, n = 3. ^*^ *p* < 0.05, ns = not significant, by one‐way ANOVA with LSD method (B, D, and F).

We next want to explore if ubiquitin‐proteasomal or the autophagic‐lysosomal pathway is involved in DBP‐induced upregulation of the DAPK3 protein. As shown in Figure S3A–D (Supporting Information), cycloheximide (CHX), a protein synthesis inhibitor, decreased the protein level of DAPK3 in TM3 cells in a dose‐ and time‐dependent manner (*P*<0.05). Moreover, the half‐life of DAPK3 was lengthened in the DBP‐treated cells (*P* < 0.05, **Figure** **6**A,B). Subsequently, MG132, a proteasome inhibitor, markedly increased the expression of DAPK3 in the cells (*P* < 0.05, Figure S3E,F, Supporting Information). Compared with the DBP‐treated cells, the protein level of DAPK3 was further increased in the MG132 plus DBP‐treated cells (*P* < 0.05, Figure 6C,D); while chloroquine (CQ), a lysosome inhibitor, had no effect on the DAPK3 protein level (*P* < 0.05, Figure 6E,F). Additionally, DBP treatment significantly inhibited the ubiquitination of endogenous and exogenous DAPK3 (Figure 6G,H). These findings confirm that DBP can inhibit ubiquitin–proteasomal degradation of DAPK3 in mouse Leydig cells.

### 2.7 DAPK3 is Ubiquitinated and Degraded by PRKN

**Figure 7:**
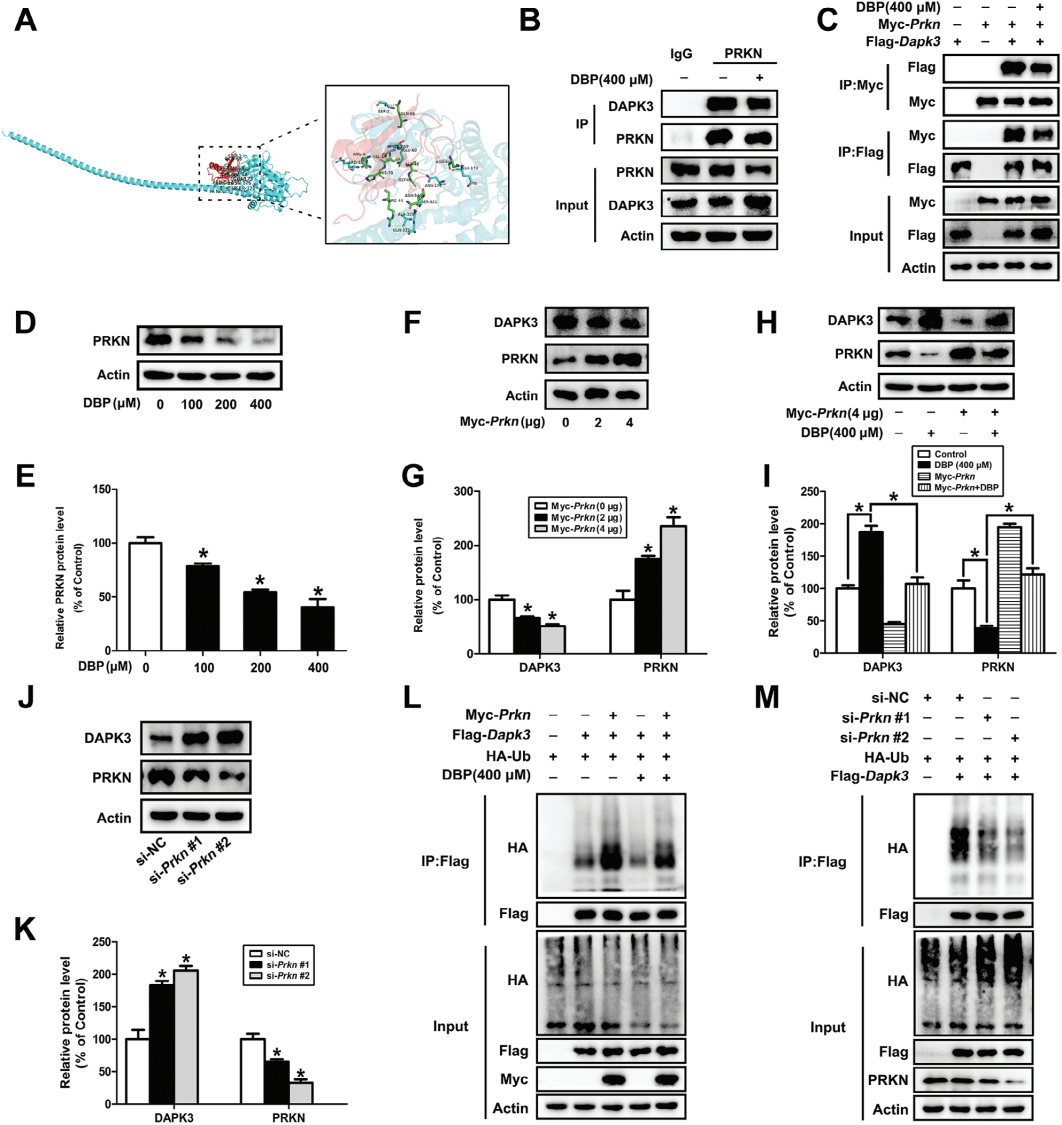
DAPK3 is ubiquitinated and degraded by PRKN. A) Surface diagram of the docking model and their interfacing residues between DAPK3 and PRKN proteins (DAPK3, blue; PRKN, red; hydrogen bond interaction, yellow dotted line). B) After TM3 cells were treated with 0 or 400 µm DBP for 24 h, and then with 50 µm MG132 for an additional 8 h, whole‐cell lysates were extracted for Co‐IP assay with IgG or anti‐PRKN antibody, and detected by Western blot with the indicated antibodies. C) TM3 cells were transfected with indicated plasmids for 48 h in the presence or absence of DBP treatment and then treated with 50 µM MG132 for another 8 h, lysates were subsequently immunoprecipitated and detected by Western blot with anti‐Flag and anti‐Myc antibodies. D,E) The protein level of PRKN was detected after TM3 cells were exposed to 0–400 µm DBP for 24 h. F,G) The expression of DAPK3 was detected after TM3 cells were transfected with 0, 2, and 4 µg Myc‐*Prkn* for 48 h. H,I) After TM3 cells were exposed to 0 or 400 µm DBP for 24 h with or without overexpression of PRKN for 48 h, the protein level of DAPK3 was determined. J,K) The protein level of DAPK3 was detected after TM3 cells were transfected with 250 pmol µL^−1^ si‐NC or two si‐RNAs targeting PRKN (si‐*Prkn* #1 and si‐*Prkn* #2) for 24 h. L) TM3 cells were transfected with indicated plasmids for 48 h in the presence or absence of DBP and then treated with 50 µm MG132 for an additional 8 h, the cell lysates were immunoprecipitated using anti‐Flag antibody and then analyzed by Western blot with indicated antibodies. M) TM3 cells were transfected with indicated plasmids for 48 h in the presence or absence of si‐*Prkn* #1 or si‐*Prkn* #2 for 24 h, and then exposed to 50 µm MG132 for an additional 8 h, the lysates were subsequently immunoprecipitated with anti‐Flag antibody and analyzed by Western blot with indicated antibodies. Data are represented as means ± SEM, n = 3. ^*^ *p* < 0.05, by one‐way ANOVA with LSD method (E, G, I, and K).

To further find out the potential E3 ubiquitin ligase ubiquitinating and degrading DAPK3 protein, we first predicted the proteins interacting with DAPK3 by HitPredict database (https://www.hitpredict.org/) and compared the predicted E3 ubiquitin ligases with the significantly downregulated DEGs. As shown in Figure S4A–C (Supporting Information), PRKN, as a primary E3 ubiquitin ligase, has the highest level of confidence and might regulate the expression of DAPK3 protein. Molecular docking showed that DAPK3 and PRKN could form hydrogen bonds through amino acid residue sites such as ASN 176‐ALA 48 and SER 321‐GLY 49, indicating that there is an interaction between DAPK3 and PRKN (**Figure** **7**A; Table S4, Supporting Information). Next, we confirmed that the reciprocal binding between DAPK3 and PRKN was weakened after treatment with DBP (Figure S4D, Supporting Information; Figure 7B,C). Meanwhile, DBP inhibited the protein content of PRKN in both testis tissue and TM3 cells (*P* < 0.05, Figure S4E,F, Supporting Information; Figure 7D,E). Then, PRKN overexpression was shown to significantly decrease the DAPK3 protein level and shorten its half‐life, which was abrogated by DBP treatment (*P* < 0.05, Figure 7F–I; Figure S5A,B, Supporting Information); while knockdown of PRKN resulted in a significant increase in the protein level and the half‐life of DAPK3 (*P* < 0.05, Figure 7J,K; Figure S5C,D, Supporting Information). Finally, PRKN overexpression led to an increase in the ubiquitination of DAPK3 protein, which was inhibited by DBP treatment; while the knockdown of PRKN markedly decreased the ubiquitination of DAPK3 (Figure 7L,M). Overall, these results suggest that PRKN can bind to DAPK3 and results in its ubiquitination and degradation in mouse Leydig cells, which is inhibited by DBP.

### 2.8 PRKN Alleviates DBP‐Induced Autophagy of Mouse Leydig Cells

**Figure 8:**
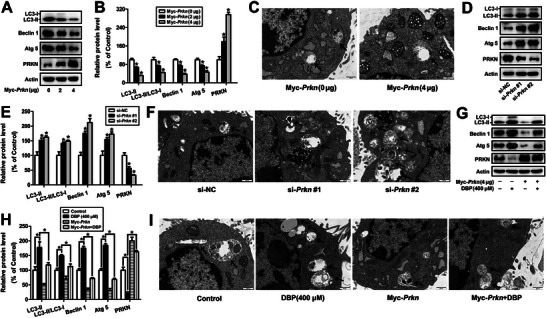
PRKN alleviates DBP‐induced autophagy of mouse Leydig cells. A–C) After TM3 cells were transfected with 0, 2, and 4 µg Myc‐*Prkn* for 48 h, autophagy was detected by Western blot and TEM, respectively. D–F) The level of autophagy in TM3 cells was detected after the knockdown of PRKN with si‐*Prkn* #1 and si‐*Prkn* #2 for 24 h. G–I) TM3 cells were transfected with 0 or 4 µg Myc‐*Prkn* for 48 h together with or without 400 µm DBP for 24 h, autophagy was then detected. Data are represented as means ± SEM, n = 3. *P < 0.05, by one‐way ANOVA with LSD method (B, E, and H).

Given that DBP can inhibit the binding of PRKN and DAPK3 and the degradation of DAPK3 in TM3 cells, so we wonder if PRKN is also involved in DBP‐induced autophagy of mouse Leydig cells. As shown in **Figure** **8**A–C, overexpression of PRKN could decrease the ratio of LC3‐II/LC3‐I, the protein contents of LC3‐II, Beclin 1 and Atg 5, as well as the amount of autophagic vesicles in TM3 cells, indicating that overexpression of PRKN inhibited autophagy of the cells (*P* < 0.05); while knockdown of PRKN by si‐RNAs led to an increase in autophagy level (*P* < 0.05, Figure 8D–F). Meanwhile, overexpression of PRKN could rescue DBP‐induced autophagy of TM3 cells (*P* < 0.05, Figure 8G–I). These findings imply that PRKN can alleviate DBP‐induced autophagy of mouse Leydig cells.

### 2.9 Melatonin Attenuates DBP‐Induced Autophagy of Mouse Leydig Cells

**Figure 9:**
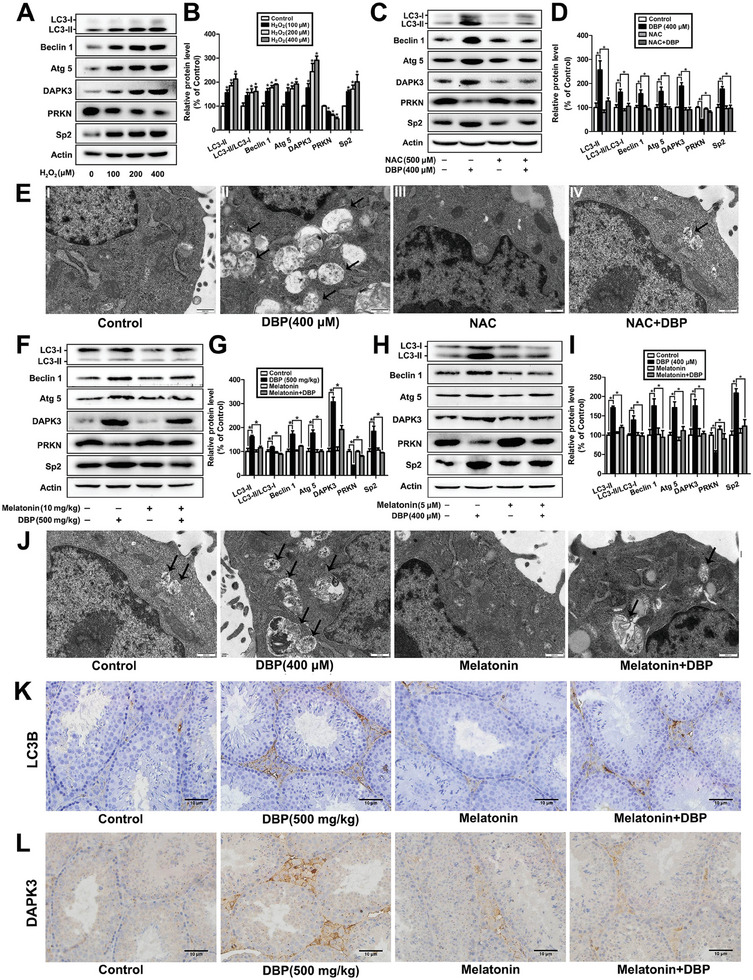
Melatonin attenuates DBP‐induced autophagy of mouse Leydig cells through regulating Sp2/DAPK3 and PRKN/DAPK3 signaling pathways. A,B) TM3 cells were treated with 0, 100, 200, and 400 µm H_2_O_2_ for 12 h, the expression of Sp2, PRKN, DAPK3, and autophagy‐related proteins were detected by Western blot. n = 3. C–E) TM3 cells were exposed to 0 or 400 µm DBP for 24 h in the presence or absence of 500 µm NAC, the protein levels of Sp2, PRKN, DAPK3, and autophagy‐related proteins, and the amount of autophagic vesicles were determined by Western blot and TEM, respectively. n = 3. F,G) After male mice were administrated with 0 or 500 mg kg^−1^ DBP in the presence or absence of 10 mg kg^−1^ melatonin for 28 d, Western blot was used to detect the protein contents of Sp2, PRKN, DAPK3, and autophagy‐related proteins in mouse testis tissue. n = 6. H–J) TM3 cells were exposed to 0 or 400 µm DBP with or without 5 µm melatonin for 24 h, the protein levels of Sp2, PRKN, DAPK3, and autophagy‐related proteins, and the number of autophagic vesicles was then determined by Western blot and TEM, respectively. n = 3. K,L) Immunohistochemistry was used to detect the expression of LC3B and DAPK3 in mouse testis tissue after male mice were gavaged with 0 or 500 mg kg^−1^ DBP in the presence or absence of 10 mg kg^−1^ melatonin for 28 d. n = 6. Data are represented as means ± SEM. *P < 0.05, by one‐way ANOVA with LSD method (B, D, G, and I).

The accumulation of reactive oxygen species (ROS) caused by oxidative stress has been confirmed to be a trigger for various programmed cell death, including autophagy, ferroptosis, and apoptosis.^[^ ^26^ ^]^ Previously, we showed that DBP can induce oxidative stress in mouse Leydig cells.^[^ ^22^ ^]^ However, it is still unknown whether oxidative stress can regulate the expression of Sp2, PRKN, and DAPK3, thereby triggering the autophagy of mouse Leydig cells. First, H_2_O_2_ (0, 100, 200, and 400 µm) and N‐acetyl‐L‐cysteine (NAC) (500 µm), an antioxidant agent, were utilized to trigger or inhibit oxidative stress of mouse Leydig cells.^[^ ^22^ ^]^ As shown in **Figure** **9**A–E, there was a significant increase in the protein levels of Sp2, DAPK3, LC3‐II, Beclin 1 and Atg 5, and the ratio of LC3‐II/LC3‐I, as well as a decrease in the protein content of PRKN in the H_2_O_2_‐treated cells, while NAC was found to abrogate DBP‐caused change in the expression of these proteins and the number of autophagic vesicles (*P* < 0.05), suggesting that DBP can affect Sp2/DAPK3 and PRKN/DAPK3 signaling pathways through oxidative stress, thereby inducing autophagy of mouse Leydig cells.

Melatonin has been shown to alleviate the damage of environmental pollutants and heavy metals to testis tissue by inhibiting oxidative stress.^[^ ^27^ ^]^ Herein, we want to determine whether melatonin can inhibit oxidative stress‐triggered Sp2/DAPK3 and PRKN/DAPK3 signaling pathways and alleviate DBP‐induced autophagy of mouse Leydig cells. As displayed in Figure 9F–J, compared with the DBP‐treated group, there was a decrease in the ratio of LC3‐II/LC3‐I, the protein levels of Sp2, DAPK3, LC3‐II, Beclin 1 and Atg 5, and the amount of autophagic vesicles, as well as an increase in the content of PRKN in mouse testis tissue and TM3 cells in the melatonin plus DBP‐treated group (*P* < 0.05). Immunohistochemistry assay indicated that melatonin could rescue DBP‐induced upregulation in the expression of LC3B and DAPK3 in mouse testis tissue, which mainly resulted from the Leydig cells (Figure 9K,L). Our findings further confirm that melatonin can regulate Sp2/DAPK3 and PRKN/DAPK3 signaling pathways by inhibiting oxidative stress, thereby alleviating DBP‐induced autophagy of mouse Leydig cells.

## 3 Discussion

In recent years, DBP has become one of the most widely used plasticizers and is often used in the manufacturing of various consumer goods such as building materials, home furnishings, and personal care products.^[^ ^28^ ^]^ DBP is commonly found in water, soil, and air, and has become a ubiquitous environmental pollutant due to its long half‐life.^[^ ^29^ ^]^ Human beings are inevitably exposed to DBP through ingestion, inhalation, and skin contact, which lead to male reproductive abnormalities by disrupting the androgen‐signaling pathway.^[^ ^30^ ^]^ DBP has also been identified to induce testicular injuries with varying degrees of severity and spermatogenesis disorders in mice.^[^ ^31^, ^32^, ^33^ ^]^ Leydig cell is the potential target for EDCs‐induced male reproductive dysfunction and its damage results in the inhibition of synthesis and secretion of testosterone.^[^ ^17^, ^34^ ^]^ We have previously confirmed that the damage to Leydig cells can be caused by DBP^[^ ^22^ ^]^; however, the underlying mechanism needs to be further studied.

Autophagy plays an extremely important role in male reproductive processes and is involved in the regulation of spermatogenesis and many pathophysiological processes of male reproductive system diseases, such as orchitis, asthenozoospermia, azoospermia, and cryptorchidism.^[^ ^35^ ^]^ Autophagy is a double‐edged sword and can maintain the survival or accelerate the death of testicular cells.^[^ ^36^ ^]^ Autophagy can also affect testosterone production and the metabolism of androgen‐binding proteins, thereby leading to spermatogenesis arrest.^[^ ^19^ ^]^ DBP can inhibit the PI3K/Akt/mTOR pathway in the genital tubercles of male offspring, thus leading to hypospadias through the induction of autophagy.^[^ ^37^ ^]^ DBP has been reported to induce autophagy in rat uroepithelial cells and genital tubercle fibroblast.^[^ ^38^, ^39^ ^]^ In this study, we found that DBP increased the protein levels of LC3‐II, Beclin 1, and Atg 5 and the ratio of LC3‐II/LC3‐I in testis tissue. Intriguingly, DBP especially increased the expression of LC3B in Leydig cells of testis tissue. As expected, DBP could also increase the levels of autophagy‐related proteins and the number of autophagic vesicles in TM3 cells. Moreover, inhibition of autophagy by 3‐MA partially rescued DBP‐caused inhibition of cell viability and activation of autophagy. These results demonstrate that DBP can induce excessive autophagy and lead to autophagic cell death in mouse Leydig cells.

To further explore the potential molecular mechanism of DBP‐induced autophagy of mouse Leydig cells, we have screened the significantly upregulated DEGs related to autophagy after TM3 cells were exposed to DBP and found there was an increase in the mRNA level of DAPK3 of the DBP‐treated cells. Subsequently, DBP was identified to promote the expression of DAPK3 in mouse Leydig cells by in vivo and in vitro experiments. As an autophagy regulator, DAPK3 can directly phosphorylate ULK1 and activate ULK‐dependent autophagy,^[^ ^40^ ^]^ and plays a crucial role in spermatogenesis via binding with T‐complex protein 10 like (TCP10L).^[^ ^41^ ^]^ Knockdown of DAPK3 in trophoblast cells blocks autophagosome‐lysosome fusion by reducing the formation of Syntaxin 17 (STX17)‐Synaptosomal‐associated protein 29 (SNAP29)‐Vesicle‐associated membrane protein 8 (VAMP8) complex.^[^ ^42^ ^]^ DAPK3 is involved in anacardic acid‐induced autophagy in prostate cancer cells^[^ ^43^ ^]^ and classical swine fever virus nonstructural protein NS5A‐induced autophagy.^[^ ^23^ ^]^ Herein, we showed that DAPK3 played an important role in DBP‐induced autophagy of mouse Leydig cells, as overexpression of DAPK3 increased autophagy, while knockdown of DAPK3 alleviated DBP‐induced autophagy.

Transcription factor Sp2 can bind to the promoters of genes regulating cell cycle, proliferation, inflammatory response, invasion, metastasis, and epithelial‐mesenchymal transition.^[^ ^44^ ^]^ Sp2 inhibits the expression of miR‐195‐5 ps by binding to its promoter region and partially promotes valvular interstitial cell calcification through the Smad‐dependent pathway.^[^ ^45^ ^]^ Sp2 can bind to the promoter region of transient receptor potential cation channel subfamily C, member 6 (TRPC6) gene, thereby promoting its transcription in mesenchymal stem cells.^[^ ^46^ ^]^ Sp2 has also been proven to be involved in lactotransferrin (LTF)‐induced autophagy of lung squamous cell carcinoma.^[^ ^47^ ^]^ We have previously found that Sp2 is upregulated in mouse Leydig cells after DBP treatment^[^ ^22^ ^]^; however, it is still unknown whether Sp2 can promote transcription of the *Dapk3* gene and is involved in DBP‐induced autophagy of mouse Leydig cells. In this study, Sp2 was found to bind to the RE3 site in the promoter region of the *Dapk3* gene and promote its gene transcription by using ChIP‐qPCR and dual‐luciferase reporter assay. Intriguingly, DBP has been identified to promote autophagy of mouse Leydig cells through the Sp2/DAPK3 signaling pathway.

Ubiquitination plays a vital role in regulating post‐translational modifications of proteins and is primarily mediated by E3 ubiquitin ligase, which specifically recognizes and ubiquitinates substrates.^[^ ^48^ ^]^ DAPK3 is a binding partner of E3 ubiquitin ligases such as ubiquitin‐protein ligase E3A (UBE3A) and LIM domain only 7 (LMO7).^[^ ^49^, ^50^ ^]^ In the current study, DBP‐caused upregulation of DAPK3 was determined to result from inhibition of ubiquitin–proteasomal degradation rather than autophagy‐lysosome degradation. Next, we determined that there was an interaction between DAPK3 and PRKN, an E3 ubiquitin ligase, and the reciprocal binding was weakened after treatment with DBP. Interestingly, knockdown of PRKN induced autophagy of TM3 cells; while overexpression of PRKN attenuated DBP‐induced autophagy. Our findings imply that DBP exposure increases the protein level of DAPK3 by inhibiting PRKN‐mediated ubiquitination and degradation, thereby inducing autophagy of mouse Leydig cells.

It is worth mentioning that PRKN has been shown to induce mitophagy, which is the selective autophagic clearance of flawed mitochondria.^[^ ^51^, ^52^, ^53^ ^]^ However, PRKN has also been reported to inhibit autophagy. For example, BCL2 mono‐ubiquitination by PRKN enhances its interaction with Beclin 1, thus inhibiting autophagy initiation; while knockdown of PRKN increases LC3 II levels.^[^ ^54^ ^]^ HIV‐1 Nef counteracts autophagy restriction by enhancing the association between Beclin 1 and BCL2 in a PRKN‐dependent manner.^[^ ^55^ ^]^ Donuts’ resistance to autophagy under depolarization is due to the prevention of mitochondria from recruiting the autophagosome receptors CALCOCO2 and OPTN even after PRKN recruitment.^[^ ^56^ ^]^ Furthermore, mitophagy still occurs in PRKN knock‐out (KO) mice.^[^ ^57^ ^]^ Therefore, the role of PRKN in regulating autophagy might be different under physiological and pathophysiological conditions, or by interacting with different substrates.

Excessive ROS‐mediated oxidative stress can cause DNA damage, ferroptosis, autophagy, and apoptosis in male germ cells, resulting in testicular dysfunction, which is also a key pathogenic factor causing male infertility.^[^ ^58^ ^]^ Exposure to phthalate esters can cause oxidative stress in testis tissue, thereby interrupting normal spermatogenesis and steroid synthesis.^[^ ^59^ ^]^ DBP‐induced oxidative stress injury in in swine testis cells could be defended against by naringenin through the PTEN/PI3K/AKT pathway.^[^ ^29^ ^]^ DBP has also been shown to induce oxidative stress in mouse Leydig cells.^[^ ^22^, ^60^, ^61^ ^]^ In the present study, we found that oxidative stress was involved in DBP‐induced autophagy of mouse Leydig cells through Sp2/DAPK3 and PRKN/DAPK3 signaling pathways. In addition, it has been demonstrated that oxidative stress can be inhibited by melatonin in Leydig cells.^[^ ^62^, ^63^, ^64^ ^]^ Melatonin, an endogenous hormone synthesized and secreted by the pineal gland, can effectively eliminate ROS,^[^ ^65^ ^]^ and inhibit high glucose or cadmium‐induced autophagy.^[^ ^66^, ^67^ ^]^ The present study confirmed that melatonin alleviates DBP‐induced autophagy of mouse Leydig cells via inhibiting oxidative stress‐triggered Sp2/DAPK3 and PRKN/DAPK3 signaling pathways.

In summary, we have first identified that DAPK3 is essential for DBP‐induced autophagy of mouse Leydig cells, and is regulated by both transcription factor Sp2 and E3 ubiquitin ligase PRKN. In addition, melatonin can alleviate DBP‐induced autophagy of the cells via inhibiting oxidative stress‐triggered Sp2/DAPK3 and PRKN/DAPK3 signaling pathways (Figure S6, Supporting Information). It cannot be denied that the exposure dose of DBP in mice used in this study is comparatively high, which is not representative of that in the general population in daily life.^[^ ^68^ ^]^ It is necessary to establish animal and cell models with chronic exposure to low doses of DBP. Meanwhile, this study mainly explored the role and mechanism of DAPK3 in DBP‐induced autophagy of mouse Leydig cells by using animal and cell experiments, while not using conditional knockout mice for *Dapk3* or *Sp2* gene. However, human exposure to DBP will increase in the future due to the fact that DBP has been widely used in the manufacture of various daily and industrial products, so the study can raise awareness to the possible outcomes of DBP exposure. Our findings have laid the groundwork in clarifying the mechanism of DBP‐induced autophagy of mouse Leydig cells, and further studies should focus on identifying DAPK3 as a potential therapeutic target for DBP‐caused male reproductive system injury.

## 4 Experimental Section

### Reagents and Antibodies

DBP (524 980, 99%), N‐acetyl‐L‐cysteine (NAC, A7250, ≥99%), 3‐methyladenine (3‐MA, M9281, ≥99%) and Melatonin (M5250, ≥98%) were provided by Sigma–Aldrich Co. LLC (St. Louis, Missouri). CCK‐8 (GK10001), cycloheximide (CHX, GC17198, >98%), proteasome inhibitor MG132 (GC10383, >98%), and chloroquine (CQ, GC19549, >98%) were obtained from GLPBIO (Montclair, America). The antibodies applied for Western blot (WB), immunohistochemistry (IHC), chromatin immunoprecipitation (ChIP) and co‐immunoprecipitation (Co‐IP) were commercially purchased as follows: Sp2 (Santa Cruz Biotechnology, sc‐17814, 1:50 for WB), Protein A/G PLUS‐Agarose (Santa Cruz Biotechnology, Sc‐2003), DAPK3 (Abmart, PK16357, 1:1000 for WB, 1:100 for IHC), Microtubule‐associated protein 1 light chain 3 (LC3, Cell signaling technology, #4108, 1:1000 for WB), Autophagy‐related gene 5 (Atg 5, Cell signaling technology, #12 994, 1:1000 for WB), β‐actin (Cell signaling technology, #4970, 1:1000 for WB), Beclin 1 (Cell signaling technology, #3495, 1:1000 for WB), Mouse IgG antibody (Proteintech, B900620), Rabbit IgG (Proteintech, B900610), LC3B (Proteintech, 18725‐1‐AP, 1:1600 for IHC), PRKN (Proteintech, 14060‐1‐AP, 1:1000 for WB), Ub (Proteintech, 10201‐2‐AP, 1:1000 for WB), HA (Proteintech, 51064‐2‐AP, 1:4000 for WB), Myc (Proteintech, 60003‐2‐Ig, 1:5000 for WB), Flag (Affinity, T0053, 1:1000 for WB) and Sp2 (Affinity, DF8721, 1:800 for IHC). TransZol Up Plus RNA Kit (ER501‐01‐V2), EasyScript One‐step gDNA Removal and cDNA Synthesis SuperMix (AE311‐02), PerfectStart Green qPCR SuperMix (AQ601‐02‐V2), TransIntro EL Transfection Reagent (FT201‐02), TransDetect Double‐Luciferase Reporter Assay Kit (FR201‐02‐V2), *Fast* Mutagenesis System (FM111‐02), and *Trans*5α Chemically Competent Cell (CD201‐01) were provided by TransGen Biotechnology (Beijing, China). Phanta Max Super‐Fidelity DNA Polymerase (P505‐02) was obtained from Vazyme (Nanjing, China).

### Animal Experiments

Kunming mice (male, 20 ± 2 g, 5 weeks old) were purchased from the Changsha Tianqin Biotechnology Co., LTD (Hunan, China). The mice were fed standard laboratory animal food and sterile water. Forty‐eight mice were randomly divided into eight groups and were subjected to intragastric administration of DBP at doses of 0, 5, 50, and 500 mg kg^−1^ day^−1^ for 28 days, or exposed to 0, or 500 mg kg^−1^ DBP for 28 days with or without intraperitoneal injection of 10 mg kg^−1^ melatonin,^[^ ^22^ ^]^ the testes were separated and collected, and then prepared for Western blot or Immunohistochemistry assay. The study was approved by the Animal Ethics Committee of Nanchang University (Approval No.NCULAE‐20220624010).

### Immunohistochemistry (IHC)

The testis tissue was fixed with neutral formalin for more than 6 h, then a series of concentrations of alcohol and xylene were used to dehydrate and make the tissue transparent, and the tissue was embedded after being immersed in paraffin to prepare 3 µm thick sections. Subsequently, the sections were deparaffinized at 70 °C for 90 min with a series of concentrations of xylene and rehydrated in ethanol. Following that, hydrogen peroxide was used to block endogenous peroxidase for 10 min. Next, the sections were incubated with anti‐DAPK3 and anti‐LC3B primary antibodies at 4 °C overnight, respectively. Then, after incubating with a secondary antibody for 30 min at 37 °C, 3′‐diaminobenzidine was utilized. Finally, hematoxylin was applied to counterstaining and a graded series of ethanol was used to dehydration, the slides were then sealed with neutral gum.

### Cell Culture

Mouse TM3 cells were cultured in DMEM/F‐12 medium supplemented with 100 IU mL^−1^ penicillin and 100 µg mL^−1^ streptomycin (Solarbio, Beijing), 5% fetal bovine serum (FBS, Excell) and 2.5% horse serum (HS, Solarbio), and maintained in a humidified incubator at 37 °C, 5% CO_2_.

### Cell Counting Kit‐8 (CCK‐8) Assay

TM3 cells were seeded in a 96‐well plate (5000 cells per well) and exposed to 0 or 400 µm DBP with or without 3‐MA (1 mm, an autophagy inhibitor) for 24 h, and 10 µL of CCK‐8 was added to each well and incubated at 37 °C for 2 h, the absorbance of each well at a wavelength of 450 nm was measured by a microplate reader.

### Western Blot Analysis

TM3 cells and testis tissue were lysed in ice‐cold RIPA lysis buffer supplemented with a protease inhibitor cocktail (Solarbio, Beijing) after exposure to the indicated treatment. BCA assay was used to quantify the concentration of protein of samples and then 5 × loading buffer was added and boiled for 10 min. Last, Western blot analysis was applied to detect the protein level in each group that contained the equal loading amount. The intensity of bands was analyzed by using the ImageJ software.

### Transmission Electron Microscopy (TEM) Analysis

TM3 cells were collected and centrifuged at 600 g for 10 min, and the supernatant was then discarded. The pellet was subsequently washed twice with ice‐cold PBS and fixed with 2.5% glutaraldehyde for 2 h. Finally, the samples were treated as described previously^[^ ^17^ ^]^ and the images were presented by TEM.

### RNA‐Sequencing

After treatment of TM3 cells with 0 or 400 µM DBP for 24 h, the total RNA was collected by Transzol Up and then sequenced according to the manufacturer's protocol provided by Shanghai Genechem Co., Ltd (Shanghai, China). Each group was independently repeated for three times. The significantly changed autophagy‐ and E3 ligase‐related genes were selected to generate heat maps with the pheatmap package by using R software.

### Construction of Plasmids, Synthesis of si‐RNAs, and Cell Transfection

The pcDNA3.1‐*Sp2* plasmid was constructed as previously described.^[^ ^22^ ^]^ The pcDNA3.1‐*Dapk3* plasmid was purchased from Fenghui Biotechnology Co., Ltd (Hunan, China). Full‐length cDNA of mouse PRKN tagged with Myc was cloned into the vector of pcDNA 3.1. The pGL4.2 vector was used to construct the pGL4.2‐*Dapk3* prom, pGL4.2‐*Dapk3* RE1 (pGL4.2‐RE1), RE2 (pGL4.2‐RE2), and RE3 (pGL4.2‐RE3) plasmids by inserting different promoter regions. The mutant plasmid of pGL4.2‐*Dapk3* RE3 (pGL4.2‐mutRE3) was produced following the manufacturer's protocol of the Fast Mutagenesis System. The primers applied for PCR are described in Table S1 (Supporting Information). The si‐*Sp2*, si‐*Dapk3*, and si‐*Prkn* were synthesized by Sangon Biotech (Shanghai, China). The siRNA sequence targeting *Sp2* is 5′‐GGAAACUGGUCCCUAUCAATT‐3′ and 5′‐UUGAUAGGGACCAGUUUCC‐3′, the siRNA sequence targeting *Dapk3* is 5′‐CAUCGCACACUUUGACCUGAATT‐3′ and 5′‐UUCAGGUCAAAGUGUGCGAUGTT‐3′, the siRNA sequence targeting *Prkn* #1 is 5′‐CGUGAUCUGUUUGGACUGUUUTT‐3′ and 5′‐AAACAGUCCAAACAGA UCACGTT‐3′, and the siRNA sequence targeting *Prkn* #2 is 5′‐CGGAGGAUGUA UGCACAUGAATT‐3′ and 5′‐UUCAUGUGCAUACAUCCUCCGTT‐3′. The above plasmids and si‐RNAs were transfected into TM3 cells according to the instructions of TransIntro EL Transfection Reagent.

### Real‐Time Quantitative PCR (qPCR) Analysis

TM3 cells were treated with the indicated concentration of DBP for 24 h, or transfected with 0, 2, and 4 µg pcDNA3.1‐*Sp2* for 48 h or 250 pmol µL^−1^ si‐*Sp2* for 24 h, or treated with 0 or 400 µm DBP for 24 h in the presence or absence of 250 pmol µL^−1^ si‐*Sp2*, or transfected with 0 or 4 µg pcDNA3.1‐*Sp2* for 48 h in the presence or absence of DAPK3 knockdown, the total RNA was isolated with Transzol Up according to the protocol of TransZol Up Plus RNA Kit. cDNAs were synthesized using EasyScript First‐Strand cDNA Synthesis SuperMix from 1 µg of total RNA and were conducted as the template in qPCR reaction which was carried out with the manufacturer's instruction of PerfectStart Green qPCR SuperMix according to CFX Connect Real‐Time System (Bio‐Rad). The primers used in qPCR analysis were designed and described in Table S2 (Supporting Information).

### Dual‐Luciferase Reporter Assay

TM3 cells were plated into 24‐well plates and co‐transfected with pRL‐TK, pGL4.2‐*Dapk3* prom/RE1‐3 or pGL4.2‐mutRE3 and pcDNA3.1‐*Sp2* or pcDNA3.1 for 48 h. After the cells were collected and lysed, the luciferase activity was analyzed by using the TransDetect Double‐Luciferase Reporter Assay Kit.

### Chromatin Immunoprecipitation (ChIP) Assay

TM3 cells were cultured in 10 cm dishes and performed by ChIP assay following the protocol as described in the previous study.^[^ ^22^ ^]^ Notably, DNA fragments were immunoprecipitated with 2 µg Sp2 antibody or mouse IgG antibody. The primers used for ChIP‐qPCR are listed in Table S3 (Supporting Information).

### Co‐Immunoprecipitation (Co‐IP)

TM3 cells were exposed to the indicated treatment, and then treated with 50 µM MG132 for 8 h before collection. First, the cells were washed with ice‐cold PBS for twice and harvested by cell lysis buffer including a protease inhibitor cocktail. Subsequently, the cells were lysed for 40 min and centrifuged at 11 000 g for 15 min at 4 °C to separate the supernatant, and 60–80 µL supernatant was taken as an input sample. The remaining cell lysates containing 500 µg‐1.5 mg proteins were incubated with indicated antibody overnight at 4 °C, and followed by incubated with 25 µL protein A/G beads for 4 h at 4 °C. Finally, the cell lysis buffer was used to wash the beads for six times, 20–30 µL 2 × protein loading buffer was then added and boiled for 10 min at 100 °C. The interaction of proteins was analyzed by Western blot analysis with the indicated antibody.

### Immunofluorescence Staining

TM3 cells were treated with 0 or 400 µM DBP for 24 h. After being washed by sterile PBS for three times, 4% paraformaldehyde was added to fix cells for 15 min at room temperature and then washed by sterile PBS once again. Next, the cells were permeabilized with PBS (0.3% Triton‐X100) for 10 min and blocked with 3% BSA (0.3% Triton‐X100). The primary antibodies against DAPK3 (1:50 dilution) and PRKN (1:200 dilution) were incubated with cells overnight at 4 °C. Similarly, the cells were washed by sterile PBS and then incubated with the goat anti‐mouse (RGAM004, proteintech, 1:200 dilution) and anti‐rabbit secondary antibody (SA00013‐2, proteintech, 1:100 dilution) for 15 min in dark at room temperature. Eventually, the antifade mounting reagent containing DAPI was applied to seal the cell slides and the images were acquired by confocal laser scanning microscope (Olympus, Japan).

### Molecular Docking Analysis

The protein structural domains of DAPK3 and PRKN were provided by the Uniprot database (https://www.uniprot.org/). GRAMM docking web server was applied to perform the rigid protein–protein docking (https://gramm.compbio.ku.edu/). PDBePISA (https://www.ebi.ac.uk/pdbe/pisa/) and Pymol software (version 3.0) were used to explore the protein‐protein interactions and further visual analysis. The results of molecular docking are presented in Table S4 (Supporting Information).

### Statistical Analysis

SPSS 19.0 was applied for statistical analysis. All data were represented as mean±standard error of the mean (SEM) and data normality was determined using the Shapiro‐Wilk tests. The comparisons between multiple groups were performed by using a one‐way analysis of variance (ANOVA), and the differences between groups were analyzed by using the least significant difference (LSD) method. The comparison between the two groups was conducted by using the independent sample *t*‐test, and *P* < 0.05 is considered to be statistically significant. Every experiment in the article was repeated in triplicate independently.

## Conflict of Interest

The authors declare no conflict of interest.

## Author Contributions

S.Y. performed in conceptualization, investigation, and writing‐original draft. Y.Y. and L.X. performed in investigation. C.H. performed in formal analysis. J.C. performed in conceptualization, writing‐review, and editing.

## Supporting information



Supporting Information

## Data Availability

The data that support the findings of this study are available from the corresponding author upon reasonable request.
