# Correction to “DAPK3 is Essential for DBP‐Induced Autophagy of Mouse Leydig Cells”

**DOI:** 10.1002/advs.202504678

**Published:** 2025-05-08

Yang S, Yang Y, Xu L, Hao C, Chen J. DAPK3 is Essential for DBP‐Induced Autophagy of Mouse Leydig Cells. Adv Sci (Weinh). 2025 Mar 6:e2413936. doi: 10.1002/advs.202413936.

In Figure 9E, the image of the transmission electron micrograph (TEM) of the NAC+DBP group appeared incorrectly. We thus request to correct Figure 9E as follows:

Corrected Figure 9E


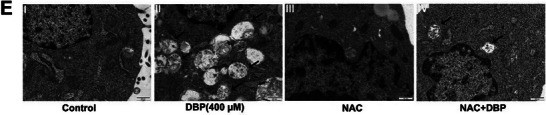


We have carefully reexamined all figures in the main document and Supporting Information, and we are confident that the correction does not impact the conclusion of our paper. We apologize for this error.

