# Supplementary material for: DAPK3 is Essential for DBP‐Induced Autophagy of Mouse Leydig Cells
Source: Adv Sci (Weinh). 2025 Mar 6;12(17):2413936. doi: 10.1002/advs.202413936 (PMC12061289; doi:10.1002/advs.202413936)

**Supplemental Fig. 1. 3-MA inhibits DBP-induced autophagy of mouse Leydig TM3 cells**. TM3 cells were exposed to 0 or 400 μM DBP for 24 h in the presence or absence of 1 mM 3-MA, cell viability **(A)**, the protein levels of LC3, Beclin 1 and Atg 5 **(B, C)** and the amount of autophagic vesicles **(D)** were then determined. The autophagic vacuoles were marked with black arrows. Data are represented as means ± SEM, n=3. **P*<0.05, by one-way ANOVA with LSD method (A, C).


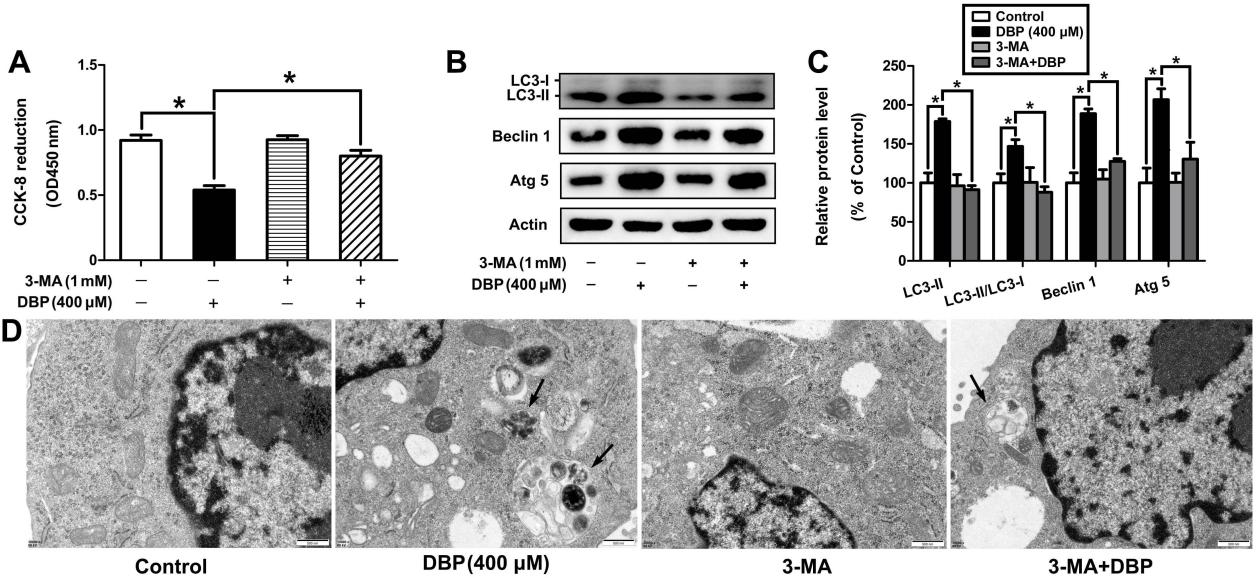


**Supplemental Fig. 2. The impact of DBP on DEGs related to autophagy in TM3 cells**. **(A)** Heat map showed a significant change in autophagy-related genes. **(B)** The mRNA levels of *Dapk3*, *Atg 5*, *Beclin 1*, *LC3B*, *Pik3r2*, *Rptor*, *Wipi2* and *Prkaca* were detected after TM3 cells were exposed to 0 or 400 μM DBP for 24 h. Data are represented as means ± SEM, n=3. **P*<0.05, by independent sample t-test (B).


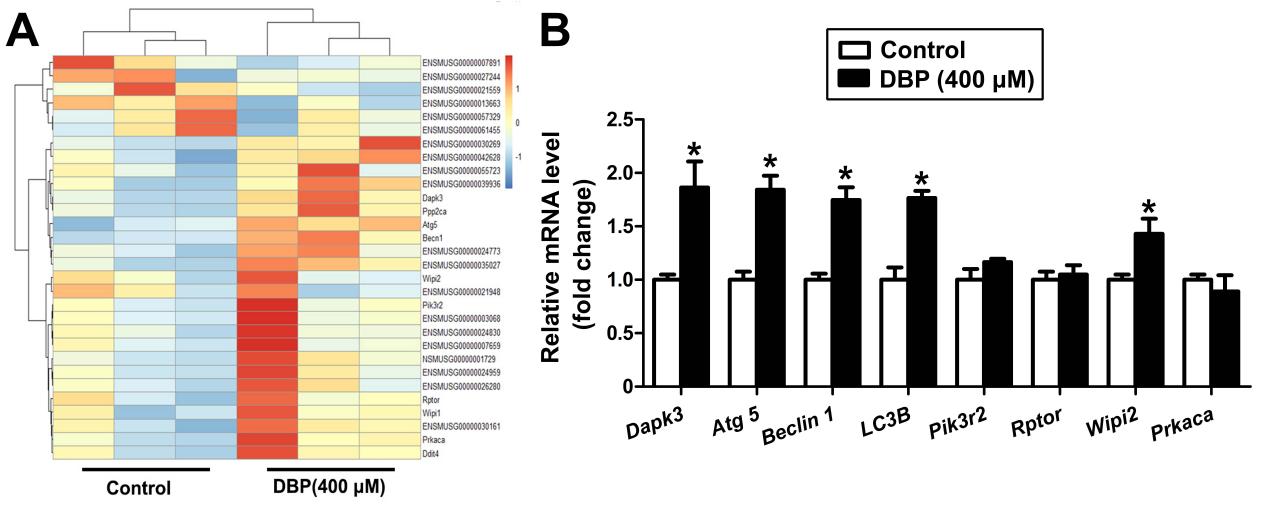


**Supplemental Fig. 3.** **DAPK3 can be degraded by ubiquitination**. **(A, B)** The content of DAPK3 protein was detected after TM3 cells were exposed to 0, 10, 20, 50 and 100 μg/mL CHX for 24 h. **(C, D)** The protein level of DAPK3 was detected after TM3 cells were exposed to 100 μg/mL CHX for 0, 6, 12 and 24 h. **(E, F)** The protein expression of DAPK3 in TM3 cells was determined after treatment with 0, 5, 10, 25 and 50 μM MG132 for 8 h. Data are represented as means ± SEM, n=3. **P*<0.05, by one-way ANOVA with LSD method (B, D and F).


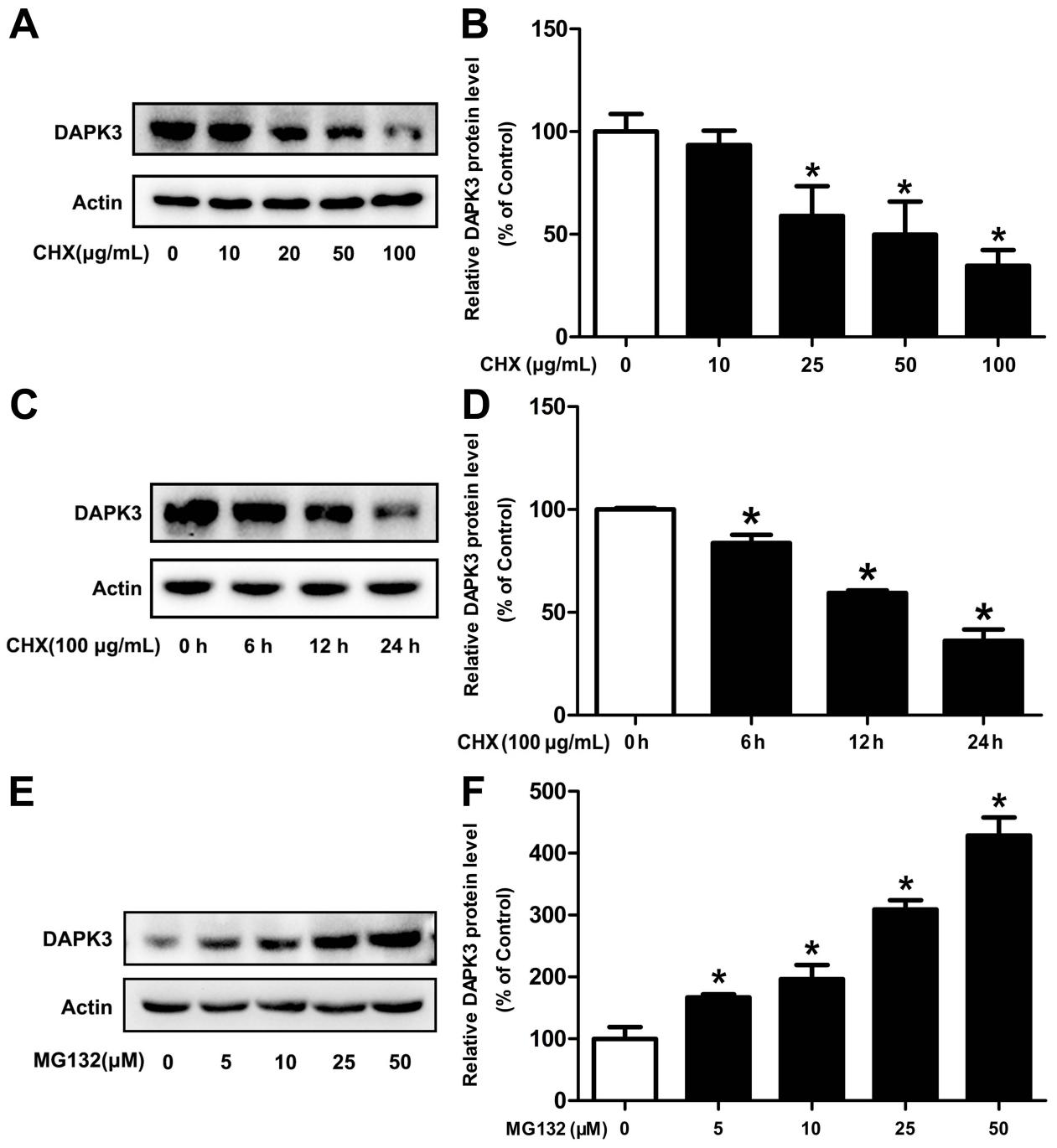


**Supplemental Fig. 4.** **Bioinformatics predicts the binding of PRKN to DAPK3**. **(A)** Heat map of significantly downregulated DEGs associated with E3 ubiquitin ligases. **(B, C)** PRKN is predicted to bind with DAPK3 by HitPredict database. **(D)** Colocalization between DAPK3 (green) and PRKN (red) in TM3 cells was weakened after treatment with 400 μM DBP for 24 h. Scale bar=10 μm. **(E, F)** After male mice were treated with 0, 5, 50 and 500 mg/kg DBP for 28 d, the protein level of PRKN was detected. Data are represented as means ± SEM, n=3. **P*<0.05, by one-way ANOVA with LSD method (F).


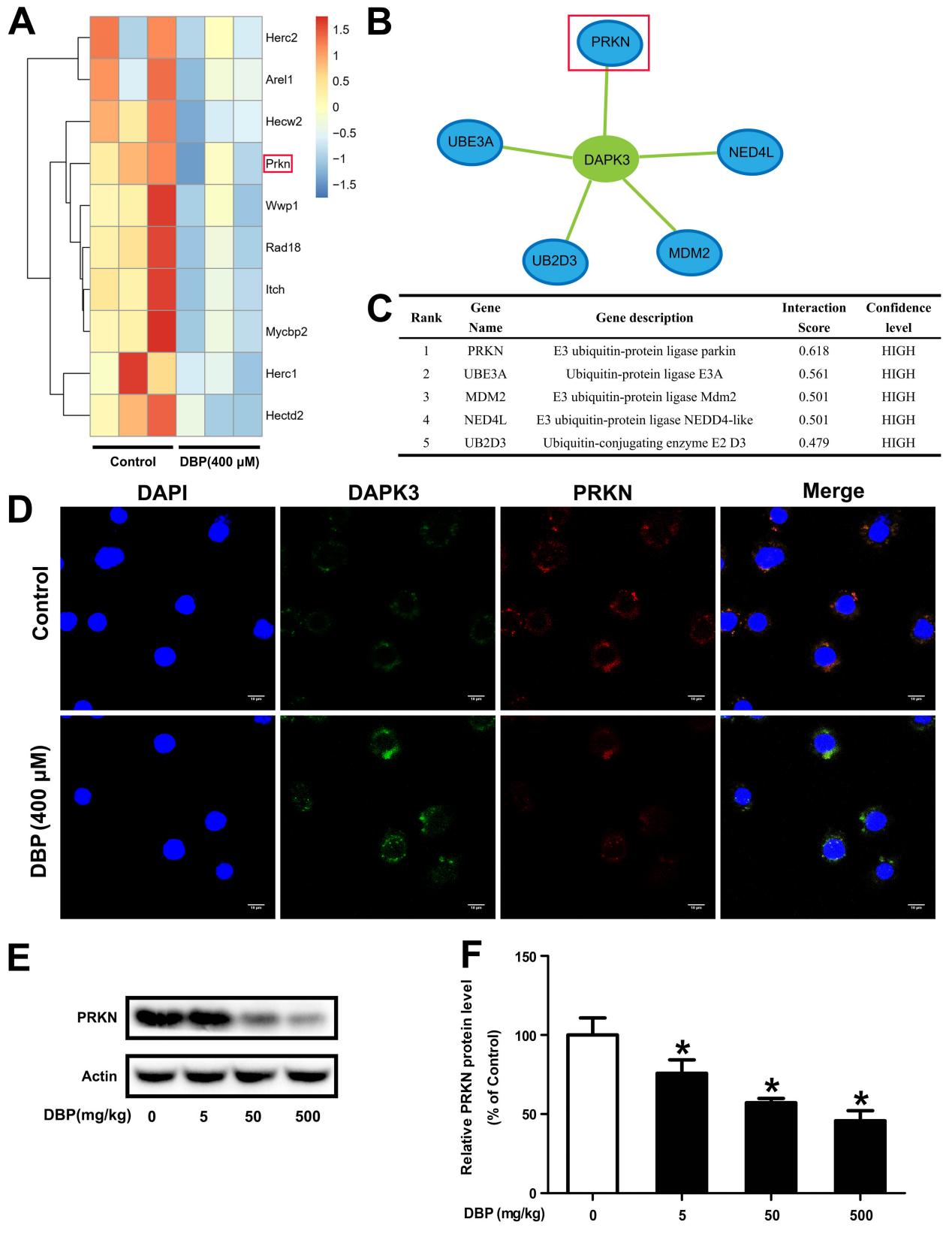


**Supplemental Fig. 5.** **PRKN promotes the degradation of DAPK3 protein**. **(A, B)** TM3 cells were transfected with 0 or 4 µg Myc-*Prkn* for 48 h in the presence or absence of 400 µM DBP for 24 h, 100 µg/mL CHX was used to treat the cells for the indicated times, and the protein level of DAPK3 was subsequently analyzed. **(C, D)** TM3 cells were transfected with 250 pmol/µL si-NC or si-*Prkn* #2 for 24 h, 100 µg/mL CHX was used to treat the cells for the indicated times, and the protein content of DAPK3 was determined. Data are represented as means ± SEM, n=3. **P*<0.05, by one-way ANOVA with LSD method (B, D).


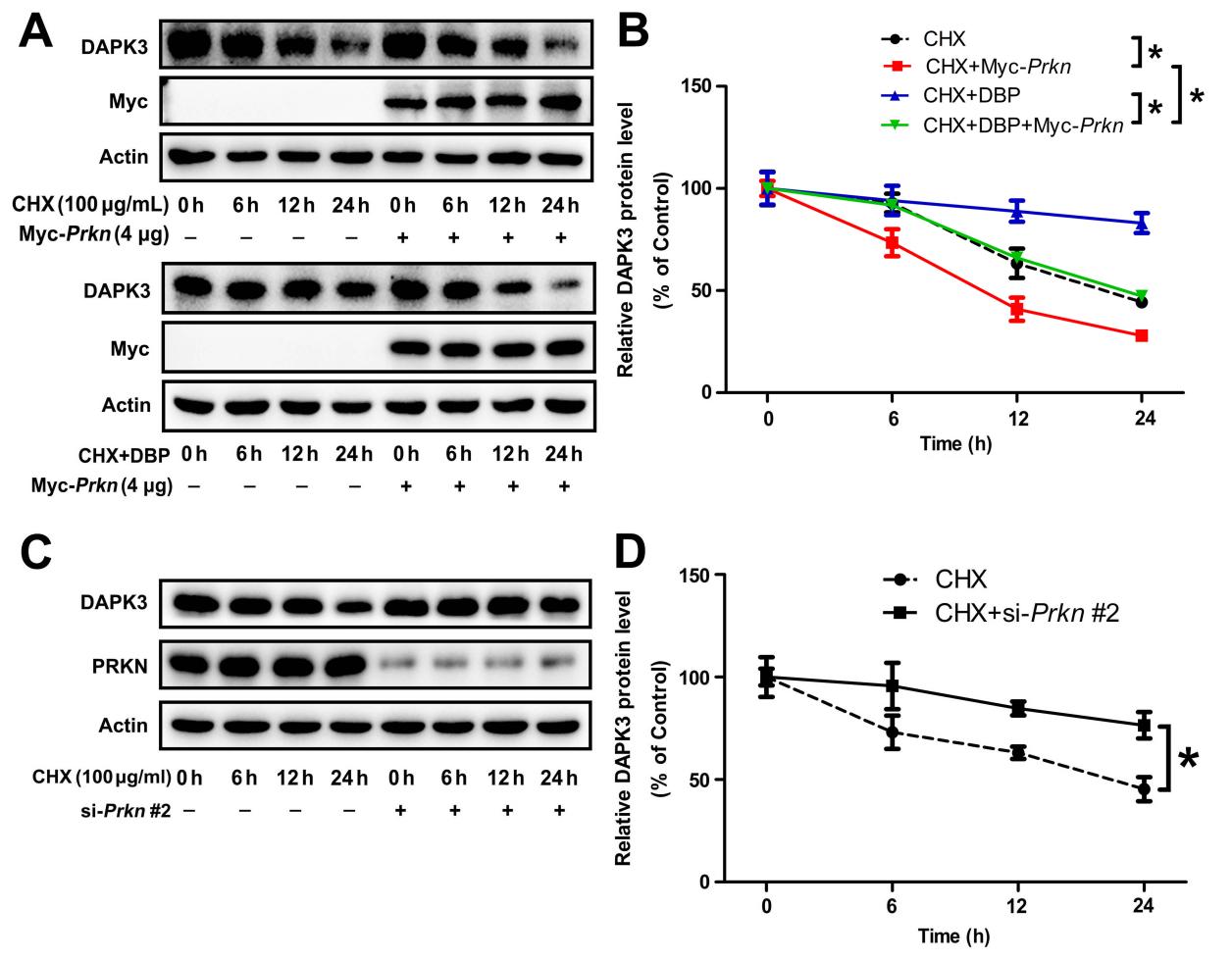


**Supplemental Fig. 6.** The molecular mechanism diagram.


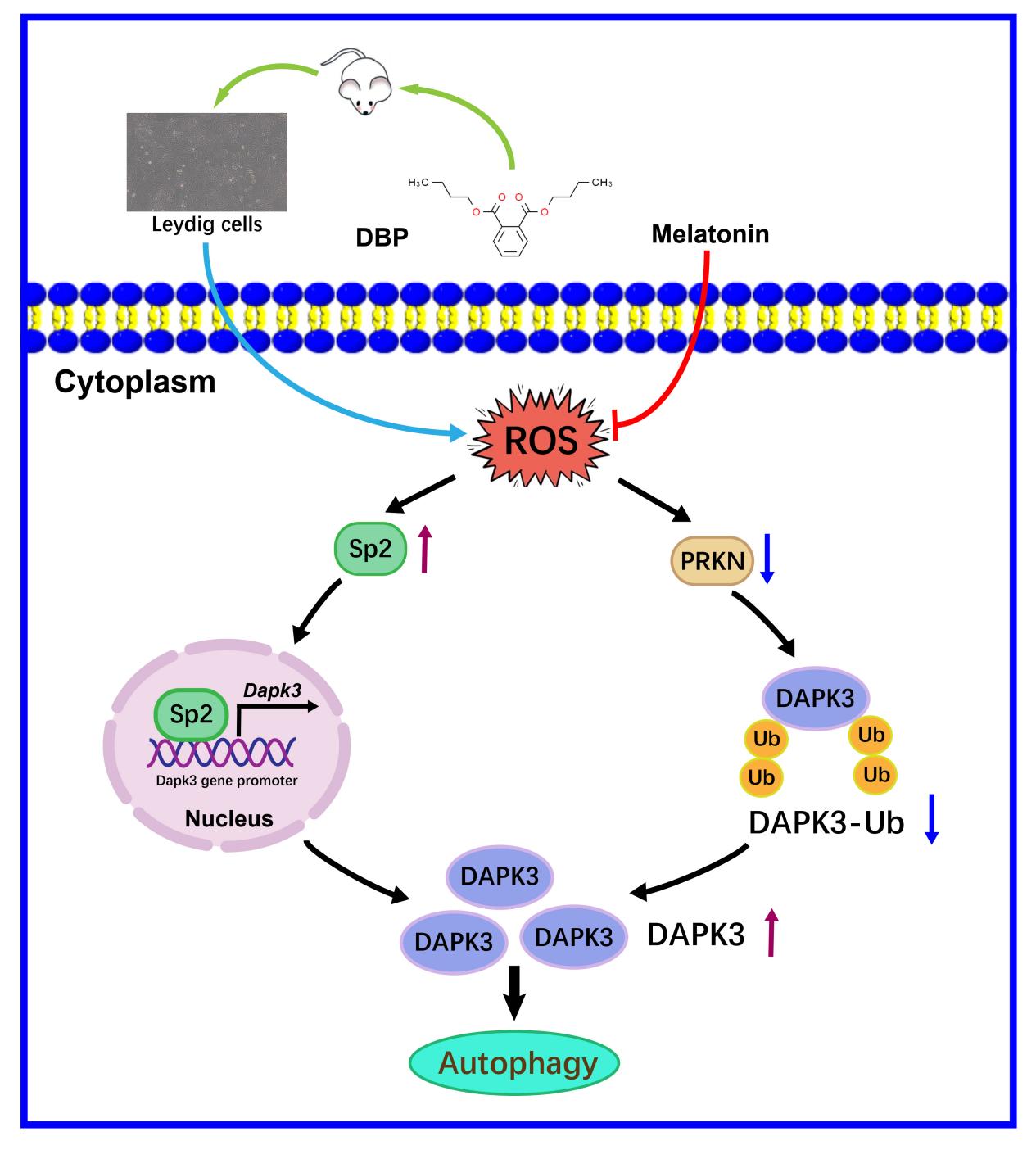


**Supplemental Table 1. The PCR primers used for constructing plasmids**


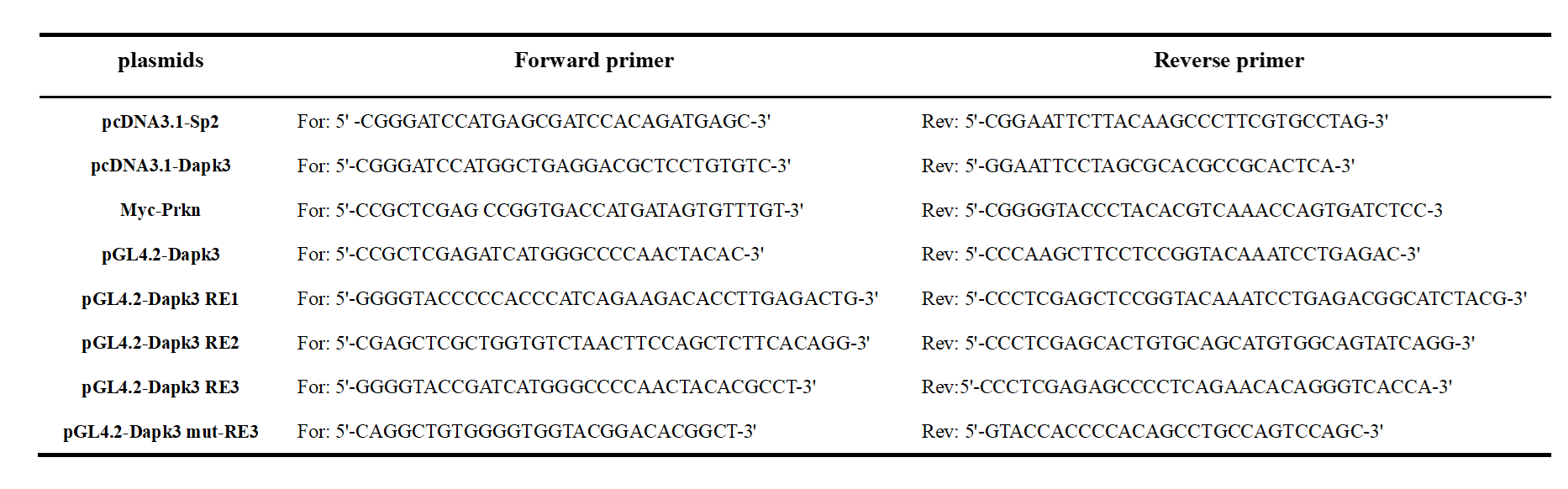


**Supplemental Table 2. Sequences of primer used in qPCR**


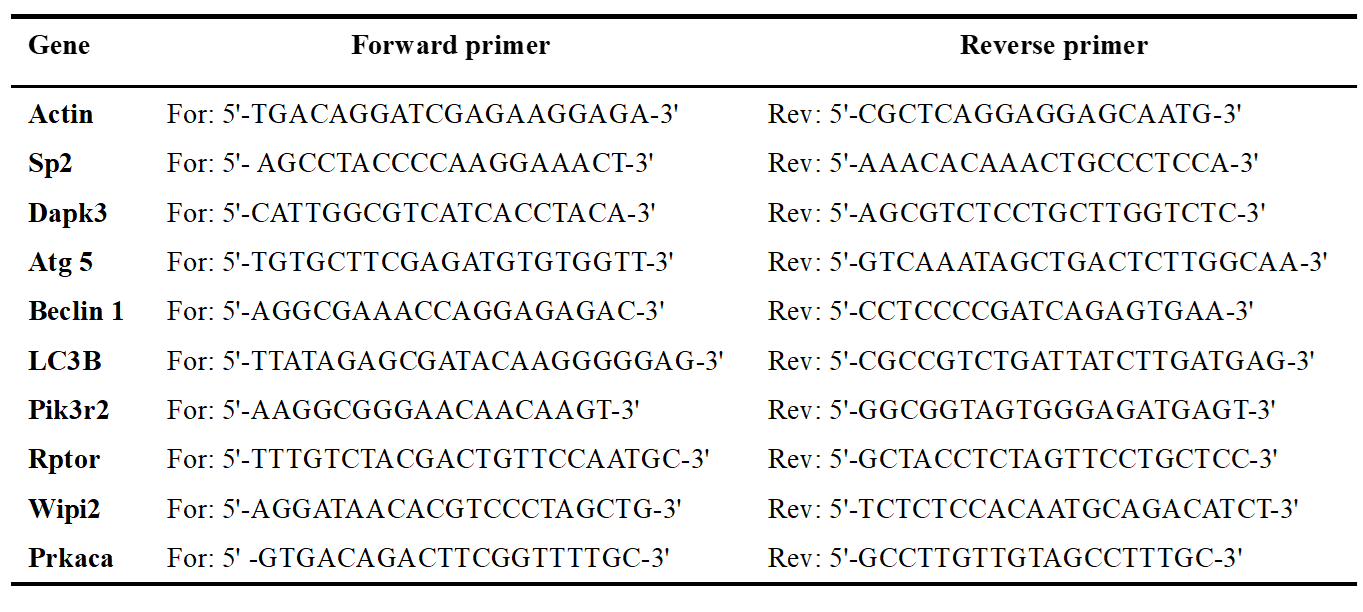


**Supplemental Table 3. The primers used for ChIP-qPCR**


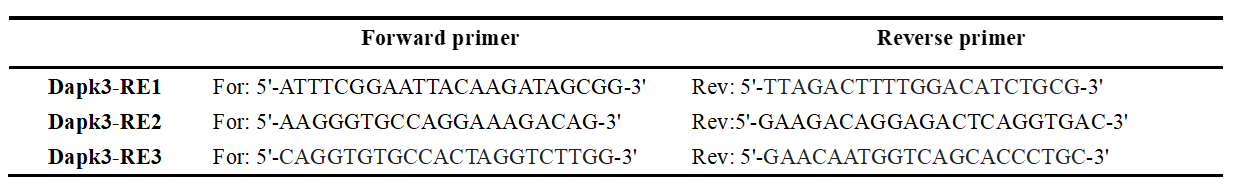


**Supplemental Table 4. The results of molecular docking**


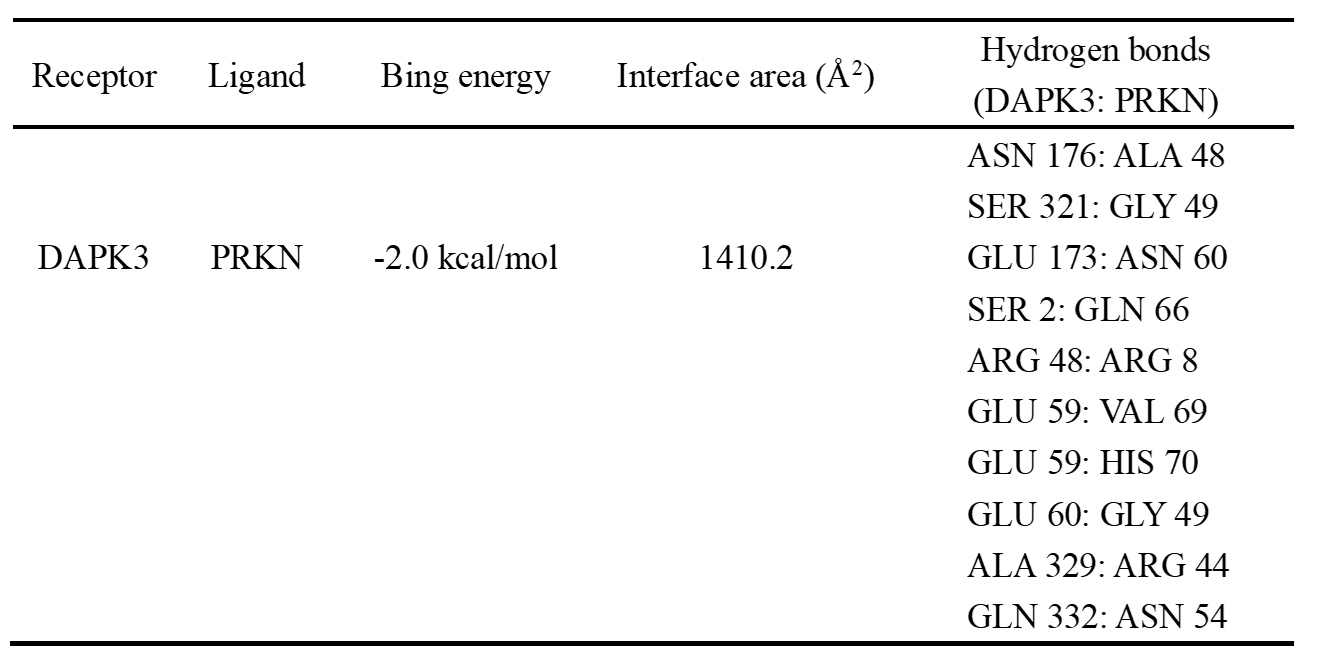

Supplement: Supplementary file 1 — Supporting Information [file ADVS-12-2413936-s001.docx]
